# Cardiopulmonary arrest in pregnancy with schizophrenia: a case report

**DOI:** 10.1186/1756-0500-7-821

**Published:** 2014-11-20

**Authors:** Takako Kudo, Akimune Kaga, Kozo Akagi, Hideki Iwahashi, Hiromitsu Makino, Yoko Watanabe, Takae Kawamura, Taiju Sato, Tsuyoshi Shinozaki, Shinya Miwa, Nobuo Okazaki, Shigeo Kure, Shingi Nakae

**Affiliations:** Division of Neonatology, National Hospital Organization Sendai Medical Center, 2-8-8 Miyagino, Miyagino-ku, Sendai, 980-8520 Japan; Department of Obstetrics and Gynecology, National Hospital Organization Sendai Medical Center, 2-8-8 Miyagino, Miyagino-ku, Sendai, 980-8520 Japan; Department of Anesthesiology, National Hospital Organization Sendai Medical Center, 2-8-8 Miyagino, Miyagino-ku, Sendai, 980-8520 Japan; Department of Cardiovascular medicine, National Hospital Organization Sendai Medical Center, 2-8-8 Miyagino, Miyagino-ku, Sendai, 980-8520 Japan; Department of Psychiatry, National Hospital Organization Sendai Medical Center, 2-8-8 Miyagino, Miyagino-ku, Sendai, 980-8520 Japan; Department of Pediatrics, Tohoku University School of Medicine, 1-1 Seiryo-machi, Aoba-ku, Sendai, 980-8574 Japan

**Keywords:** Cardiopulmonary arrest, Hypoxic-ischemic encephalopathy, Multidisciplinary approach, Resuscitation, Schizophrenia

## Abstract

**Background:**

Cardiopulmonary arrest in pregnancy has a very high maternal and fetal mortality rate. We report a case of successful maternal and neonatal survival in association with emergency cesarean section of a schizophrenic pregnant patient. To our knowledge, this is the first reported case of cardiopulmonary arrest in a pregnant woman with schizophrenia.

**Case presentation:**

The parents were Japanese. The mother was 39 years old and had no history of prior pregnancy. Her admission to our hospital at 36 weeks and 4 days of pregnancy was due to deterioration of schizophrenia. On the first day of hospitalization, she collapsed after a seizure and vomiting, and an emergency resuscitation team was called immediately. The team identified apparent aspiration and successfully resuscitated the patient after 11 minutes of cardiopulmonary arrest. An emergency cesarean section was performed in the operating room. The newborn male infant received bag and mask ventilation at birth, and his Apgar scores were 5 at 1 minute and 8 at 5 minutes. He had a myoclonic seizure on the 2nd day of life: however, he experienced no further seizures on anticonvulsant medication after that episode. On the 18th day of life, magnetic resonance imaging of his brain revealed bilateral small hyperintensities on T_1_-weighted images in the basal ganglia. The mother and her newborn were discharged from our hospital without neurological disorders.

**Conclusion:**

We speculate that the cause of cardiopulmonary arrest was aspiration due to seizure, and it is possible that a neurological response was evoked by administration of antipsychotic drugs and/or by eclampsia. Medical staff must be aware of the possibility of cardiopulmonary arrest in pregnant women with schizophrenia.

## Background

Cardiopulmonary arrest in pregnancy carries a very high maternal and fetal mortality rate [1], with an incidence estimated at 1 in approximately 25,000 to 30,000 ongoing pregnancies [2, 3]. Several studies have reported an association between cardiopulmonary arrest in pregnancy and maternal disease [1–9]. In this article, we report a case of successful maternal and neonatal survival in association with emergency cesarean section. To our knowledge, a case of cardiopulmonary arrest in a pregnant woman with schizophrenia has not previously been reported.

## Case presentation

**Figure 1:**
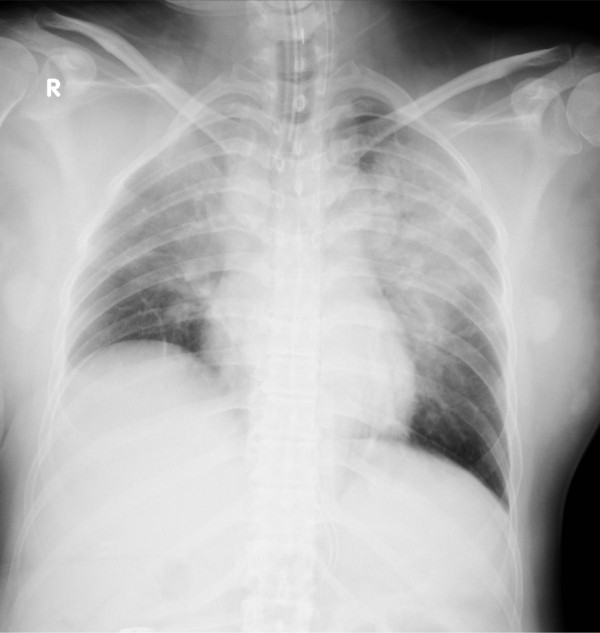
**A chest X-ray of the mother reveals consolidation at bilateral upper lung field.**

The parents were Japanese. The mother was 39 years old, with no history of prior pregnancy. She had suffered from schizophrenia since the age of 19 years. At 36 weeks and 4 days of pregnancy, she was admitted to National Hospital Organization Sendai Medical Center owing to a relapse of schizophrenia. On admission, she was agitated and her speech was incoherent. Her heart rate and blood pressure were 135 beats/min and 154/91 mmHg, respectively. Her blood cell counts were as follows: leukocytes, 8,900/μL (neutrophils 82.1%, lymphocytes 11.7%, monocytes 5.7% and eosinophils 0.3%); red blood cells, 4.69 × 10^6^/μL; hemoglobin, 13.0 g/dL; hematocrit, 39.0%; and platelets, 321,000/μL. Laboratory data, including levels of glucose, hepatic enzymes, coagulants, creatine kinase and creatine, were normal, but the level of uric acid was high (7.6 mg/dL). No evidence of an inflammatory reaction or serum electrolyte abnormalities was observed. A chest X-ray and electrocardiogram showed no abnormal findings. She received an oral dose of 6 mg of risperidone, 150 mg of quetiapine, 7.5 mg of olanzapine and 1 mg of etizolam instead of the previously administered 48 mg of perospirone. That night, she experienced a generalized tonic seizure, which was controlled by intramuscular injection of 10 mg of diazepam. After 6 minutes, another generalized tonic seizure occurred, and magnesium sulfate was intravenously injected as a treatment for eclampsia. After 14 minutes, she vomited a large amount of food, and her oxygen saturation level soon dropped to 50% with bradycardia. Her pulse could not be detected, and a cardiopulmonary resuscitation team was immediately called. The team found that food was present in her airway at the time of intubation, and they successfully resuscitated her after 11 minutes of cardiac arrest. During the seizures, neither ventricular fibrillation nor torsade de pointes was observed. A computed tomography scan showed no cerebral hemorrhage and no pulmonary embolism. At the time of 1 hour and 7 minutes after her cardiopulmonary arrest, an emergency cesarean section was performed in the operating room. After the operation, she was transferred to the intensive care unit (ICU), where she received hypothermia therapy to minimize brain damage. An electrocardiogram was normal. A chest X-ray revealed consolidation at the bilateral upper lung field (Figure 1). She received intravenous antibiotics for suspected aspiration pneumonia. Laboratory data, including levels of glucose, creatine kinase and cardiac enzymes, were normal. After 3 days, the patient was lucid, and she left the ICU. On the 13th day after admission, an electroencephalogram was normal. She was discharged without neurological disorders on the 51st day after admission.

**Figure 2:**
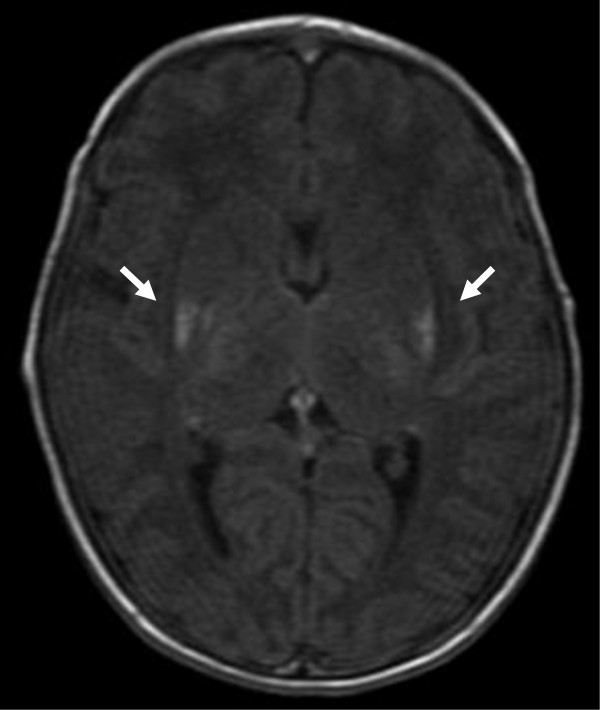
**Brain head magnetic resonance reveals bilateral small hyperintensities on T1-weighted images in the basal ganglia: bilateral putamen, pallidum and thalamus (white arrow).**

The newborn was a male with a birth weight of 2568 g. He received bag and mask ventilation at birth, and his Apgar scores were 5 at 1 minute and 8 at 5 minutes. The pH and base excess of his umbilical arterial blood gas analysis were low (7.046 and −13.4 mEq/L, respectively). He was admitted to our neonatal intensive care unit. The Moro, tendon, grasp and light reflexes were normal and the muscle tonus was normal. He did not receive therapeutic hypothermia due to his neurological findings (Sarnat stage I) on admission and immediately improvements of his neurological findings, general condition and blood gas data, which were not suitable to Japanese manual of hypothermia therapy for newborn infant [10]. On the 2nd day of life, he experienced a myoclonic seizure. No evidence of inflammatory reaction or serum electrolyte abnormalities, including calcium or coagulation disorders, was observed. Ultrasonography showed no intracranial hemorrhage. On the 7th day of life, electroencephalography showed sharp waves and single spikes occurring in the temporal or central region. On the 18th day of life, brain magnetic resonance imaging (MRI) revealed bilateral small hyperintensities on T_1_-weighted images in the basal ganglia (Figure 2). He had no seizures on anticonvulsant medication with phenobarbital. On the 37th day of life, he was discharged from our hospital. At 4 months of age, the patient exhibited favorable growth and development, and had no further seizures with continuous oral phenobarbital treatment.

## Discussion

The major causes of cardiopulmonary arrest in pregnancy are hemorrhagic, septic or anaphylactic shock; trauma; pulmonary or amniotic fluid embolism; and congenital or acquired cardiac disease [4, 5]. Cardiac disease is the leading cause of maternal mortality based on a United Kingdom database that holds the largest population-based data on this specific group [8]. In this case, although we suspected that the cause of cardiopulmonary arrest was long QT interval syndrome or torsade de pointes associated with the administration of antipsychotic drugs, an electrocardiogram showed no abnormality. Cerebral hemorrhage or pulmonary embolism was considered unlikely because a computed tomography scan also showed no abnormality. We speculate that the cause of cardiopulmonary arrest was aspiration due to seizure, and it is possible that a neurological response was evoked by administration of antipsychotic drugs and/or by eclampsia. In Japan, the prevalence of schizophrenia was relatively high and the number of schizophrenics was increasing [11]. We speculate that the number of Japanese pregnant woman with schizophrenia will also increase in Japan in the future. We conclude that medical staff must be aware of the possibility of non-specific symptoms including cardiopulmonary arrest in pregnant women with schizophrenia.

Cardiopulmonary arrest in pregnancy is a rare but terrifying situation, with maternal and neonatal fatality rates of 83% and 58%, respectively [1]. Anoxic brain injury occurs within 4 minutes after a cardiopulmonary arrest is identified [9]. In this case, the patient was able to achieve return of spontaneous circulation after 11 minutes. We performed computed tomography to evaluate cerebral hemorrhage and pulmonary embolism. The patient delivered via cesarean delivery at approximately 1 hour and 12 minutes after cardiopulmonary arrest. Although we took the time to investigate the cause of cardiopulmonary arrest, we might have performed an emergency cesarean section immediately after the cardiopulmonary arrest.

Hypoxic-ischemic encephalopathy, usually secondary to perinatal asphyxia, is the single most common cause of neonatal seizures in both full-term and premature infants [12]. Factors related to the severity and the temporal characteristics of the insult appear to be of particular importance in determining the major pattern of selective neuronal injury in the newborn [13]. The cerebral-deep nuclear pattern of neuronal injury appears to be related to insults that are less severe and prolonged, often termed partial, prolonged asphyxia [13]. The deep-thalamus-brain stem pattern of injury to basal ganglia-thalamus-brain stem has been described with a severe, abrupt event, often termed total asphyxia [13]. In this newborn infant, a brain MRI revealed bilateral small hyperintensities on T_1_-weighted images in the basal ganglia, which suggested total asphyxia. We speculated that total disruption of cerebral blood flow had occurred due to cardiac arrest-induced severe hypotension. It is postulated that a severe, abrupt event hampers the function of the major adaptive mechanisms that normally operate during an asphyxia event. The most important of these mechanisms may be diversion of blood from the cerebral hemispheres to the vital deep nuclear structures [13]. Because the latter have high rates of energy use, these nuclei are particularly likely to be injured [13]. Fortunately, the lesion of our case did not extend to the caudate nucleus or brainstem. After the newborn infant’s generalized tonic seizure on the 2nd day of life, he had no further seizures on anticonvulsant medication. Although he has not shown any signs of neurological impairment, careful follow-up is mandatory for the possibility of presence of delayed effects such as motor impairment, cognitive deficits and dystonia [14].

Cardiopulmonary arrest during pregnancy presents unique clinical effects on involving two patients: the mother and the fetus. Management of these patients demands a rapid multidisciplinary approach including the obstetrician, neonatologist, anesthesiologist and cardiologist [9]. In our institution, obstetricians, neonatologists, anesthesiologists, maternity nurses and operating room nurses have routinely performed simulation training for emergency cesarean (we refer to this as grade A cesarean section). However, in this case, because the mother had a complicated disease and subsequently developed cardiac arrest, we needed to cooperate with other medical specialists and facilities, including a cardiologist, a psychiatrist, an emergency unit and an ICU. We conclude that a multidisciplinary approach involving cooperation among related divisions, as well as frequent simulation training, is important to address such cases.

## Conclusion

We speculate that the cause of cardiopulmonary arrest was aspiration due to seizure, and it is possible that a neurological response was evoked by administration of antipsychotic drugs and/or by eclampsia. Medical staff must be aware of the possibility of cardiopulmonary arrest in pregnant women with schizophrenia.

## Consent

Written informed consent was obtained from the patient (the mother) for publication of this Case Report and any accompanying images. A copy of the written consent is available for review by the Editor-in-Chief of this journal.

## Abbreviations

ICU: Intensive care unit
MRI: Magnetic resonance imaging.
